# Inability and Obligation in Moral Judgment

**DOI:** 10.1371/journal.pone.0136589

**Published:** 2015-08-21

**Authors:** Wesley Buckwalter, John Turri

**Affiliations:** 1 Department of Philosophy, University of Waterloo, Waterloo, Ontario, Canada; 2 Cognitive Science Program, University of Waterloo, Waterloo, Ontario, Canada; University of Vienna, AUSTRIA

## Abstract

It is often thought that judgments about what we ought to do are limited by judgments about what we can do, or that “ought implies can.” We conducted eight experiments to test the link between a range of moral requirements and abilities in ordinary moral evaluations. Moral obligations were repeatedly attributed in tandem with inability, regardless of the type (Experiments 1–3), temporal duration (Experiment 5), or scope (Experiment 6) of inability. This pattern was consistently observed using a variety of moral vocabulary to probe moral judgments and was insensitive to different levels of seriousness for the consequences of inaction (Experiment 4). Judgments about moral obligation were no different for individuals who can or cannot perform physical actions, and these judgments differed from evaluations of a non-moral obligation (Experiment 7). Together these results demonstrate that commonsense morality rejects the “ought implies can” principle for moral requirements, and that judgments about moral obligation are made independently of considerations about ability. By contrast, judgments of blame were highly sensitive to considerations about ability (Experiment 8), which suggests that commonsense morality might accept a “blame implies can” principle.

## Introduction

Morality is central to human social life [1–3]. Fulfilling moral obligations often requires us to put other people’s interests before our own. Sometimes this is easy, but other times it is hard. For example, it is plausible we are obligated to alleviate terrible suffering if we can do so at very little cost to ourselves, as happens when we donate money to famine relief or vaccination programs. But how far does this obligation extend? Some argue that it extends to the point where we would be making ourselves worse off than the people receiving charitable aid [4]. Many have found this suggestion implausible, sometimes on the grounds that the requirements for morality are limited by our psychology [5–7]. Given the way we are constituted, perhaps we are simply incapable of donating that much. This raises an important question: how demanding is morality and what are the limits of moral requirements?

According to a longstanding principle of moral philosophy, moral requirements are limited by *ability*. This is often glossed by the slogan that “ought implies can” (hereafter “OIC” for short). The principle says that one is obliged to perform an action only if one can perform the action. Support for OIC can be traced back to at least Cicero [8]. A more explicit articulation comes from Immanuel Kant, who writes, “Duty commands nothing but what we can do,” and that, “If the moral law commands that we ought to be better human beings now, it inescapably follows that we must be capable of being better human beings” [9].

Contemporary philosophers widely endorse various versions of OIC concerning what one is obliged, ought, or has duties to do when one is unable to do it [10–21]. The principle is also frequently invoked in debates in moral philosophy over the relationship between moral obligations and alternative possibilities [10, 22–25], and the relationship between determinism and free will [26–28]. OIC is also often invoked in discussions about the possibility of genuine moral dilemmas, such as classic “trolley problem” thought experiments [20, 29–32]. Some have even claimed that it is a principle of commonsense morality that laypeople accept [33]. Though there are some notable exceptions [18, 33], there is widespread agreement about OIC and it continues to remain an influential principle of moral philosophy.

Over the past decade, psychologists have made many important discoveries about moral cognition. Many of these occurred because psychologists took seriously longstanding discussions in moral philosophy about certain theories, principles, and thought experiments. For example, examining intuitive reactions to trolley problems led to advancements in dual-process models of moral cognition [34–36]. Studying intuitive reactions to the philosophical problem of “moral luck” has helped distinguish the causal and intentional properties contributing to moral judgment [37, 38]. Similarly, other psychological discoveries have been informed by philosophical contributions to the theory of intentionality [39, 40], objectivism [41], personhood [42], and ownership [43]. In the present paper, we follow a similar path. The main difference is that our starting point is OIC and our goal is to understand the relationship between attributions of ability, moral requirements, and blame.

Prior research in psychology has demonstrated that judgments about what agents *will* do are strongly influenced by perceptions of their ability to perform them [44–47]. It is currently unknown whether perceptions of ability also influence judgments about what agents morally *ought* to do, although two existing lines of evidence lend mild support for this claim. First, researchers have found that inability diminishes people’s willingness to punish an agent. For example, in resource allocation games, people punish greedy allocators significantly less when the allocator is unable to select a generous allocation [48–52]. Perhaps this is because such allocators are less likely to be judged as morally obligated. That is, if they are not able to act generously, then perhaps they are not obligated to act generously and, thus, not to be punished for acting otherwise. Second, similar reactions are found in assessments of legal liability for uncontrollable actions. For example, perpetrators of violent crimes are thought to deserve much less punishment or criminal liability when their actions are due to cognitive dysfunctions or brain tumors [53, 54]. This also suggests that people make allowances by lowering expectations for the moral requirements of behaviors given that they are less likely to be perceived as able to control them.

While these observations might be suggestive, the relationship between judgments of ability and morality has not been studied directly. This paper reports eight experiments that study it directly. Our primary question is whether perceptions of ability limit judgments about whether a broad range of moral requirements are present, or if moral requirements are attributed to agents independently of the presence of an ability to fulfill them. The experiments focus on straightforward cases featuring ordinary agents and moral decisions frequently encountered in everyday life. They also focus on familiar ways that agents are unable to act, due to a variety of common physical and psychological inabilities. We use a variety of different moral terminology to evaluate support for the OIC principle, or something thereabout, in ordinary moral cognition, including “ought”, “obligation”, and “duty”.

The main findings are that, contrary to prevailing philosophical wisdom, commonsense morality rejects the “ought implies can” principle. Moral requirements were attributed independently of ability, regardless of the type (Experiments 1–3), temporal duration (Experiment 5), or scope (Experiment 6) of inability. This result was consistently observed using a range of moral vocabulary to probe moral judgments and was insensitive to different levels of seriousness for the consequences of inaction (Experiment 4). Judgments about moral obligation were no different for individuals who can or cannot perform physical actions (Experiment 7). By contrast, attributions of blame were highly sensitive to considerations about ability (Experiment 8), which suggests that commonsense morality might accept a “blame implies can” principle. We discuss the implications of these findings for moral psychology, moral philosophy, and related questions in mental health research, criminal justice, and jurisprudence.

## Experiment 1

We should honor our promises, but sometimes we make promises that we cannot keep. Philosophers have wondered whether there can be genuine obligations to honor promises in such circumstances given the “ought implies can” principle [55, 56]. For instance, suppose that an agent makes a promise to meet Brown but is physically or psychologically unable to keep the appointment [56]. The first experiment uses this case from the ethics literature to test whether such agents are considered obligated to meet Brown.

### Method

#### Participants

Eighty participants were tested (aged 18–59 years, mean age = 32.35 years; 91% reporting English as a native language; 21 female). Participants were recruited and tested using an online platform (Amazon Mechanical Turk [AMT] and Qualtrics) and compensated $0.30 for approximately 2 minutes of their time. Written informed consent was given prior to online participation. Ethics clearance to conduct this research with human participants was granted by the University of Waterloo Office of Research Ethics. Participation was restricted to United States residents. Participants were not allowed to re-take the study. Repeat participation, within and across experiments, was prevented by screening AMT Worker IDs. We decided in advance to test 40 participants per condition, which dictated the sample size for this and all subsequent experiments reported in the paper. These same basic recruitment and compensation procedures were used in all other experiments reported below.

#### Materials and procedure

Participants were randomly assigned to one of two conditions in a between-subjects design. Participants in each condition read a single story. The stories all featured an agent, Walter, who promises to pick up Brown at the airport. In each story, Walter lacks the ability to pick up Brown at the airport on the day of Brown’s flight [56]. The stories differed in Walter’s type of inability. In the Physical condition, Walter is physically unable to pick up Brown. In the Psychological condition, Walter is psychologically unable to do so.

Walter promised that he would pick up Brown from the airport. But on the day of Brown’s flight, Walter is [in a serious car accident/suffering from clinical depression]. As a result, Walter is not [physically/psychologically] able to pick up Brown at the airport.

After reading a story, participants were asked the same set of questions. The first question was designed to test the “ought implies can” link. The OIC test question asked participants to select whether Walter is obligated or not obligated to pick up Brown, on the one hand, and whether Walter was physical or psychologically able to do so, on the other. This task was designed to force participants to consider obligation and ability simultaneously, which is preferable to individual evaluation due to potential biases that may occur when attributing them in isolation [57]. In the Physical condition, for instance, participants were asked, “Please choose the option that best applies” from the responses below:

1. Walter is obligated to pick up Brown at the airport, and Walter is physically able to do so.
2. Walter is obligated to pick up Brown at the airport, but Walter is not physically able to do so.
3. Walter is not obligated to pick up Brown at the airport, but Walter is physically able to do so.
4. Walter is not obligated to pick up Brown at the airport, and Walter is not physically able to do so.

The order of all response options was randomized. (Participants never saw the numerical labels.) After answering the OIC test question, participants were taken to a new screen with two additional questions. They were first presented with an inability probe, “Walter is literally unable to pick up Brown at the airport”. The purpose of the inability probe was to check if participants thought that the physical or psychological inability rendered the agent truly unable to fulfill the obligation. Responses were collected on a yes/no dichotomous scale. They were then asked to rate their agreement or disagreement with the blame item “Walter deserves to be blamed for the fact that Brown was not picked up.” Responses for the blame item were collected on a standard Likert scale, 1 (“Strongly disagree”)– 7 (“Strongly agree”). Participants could not go back and change their answers. The story remained at the top of the screen throughout. After testing, participants completed a brief demographic survey.

### Results

Preliminary analysis revealed no effects of participant age, gender, or native language on the dependent variables, so the analyses that follow collapse across those demographic variables. The same is true in all the other experiments reported in this paper.

Assignment to condition did not affect responses to the OIC test question, X^2^ (3, *N* = 80) = 2.81, *p* = .422. Participants selected the “Obligated but Unable” response in both Physical and Psychological conditions, 80%/88%, which each exceeded chance rates, binomial test, *p*s < .000001, test proportion = .25.

Assignment to condition did affect the extent to which participants agreed that the protagonist was literally unable to pick up Brown, with 100% answering “yes” in the physical condition, and 32% answering “yes” in the psychological condition, X^2^ (1, *N* = 80) = 40.98, *p* < .000001. The magnitude of this difference was extremely large, Cramer’s V = .716. Also, mean agreement with how much blame the protagonist deserves was significantly lower in the Physical condition (*M* = 1.95, *S D* = 1.08) than in the Psychological (*M* = 4.49, *SD* = 1.76) condition; *t*(78) = -7.73, *p* < .000001, η_p_^2^ = .434.

To further evaluate OIC, responses to the OIC test question were reanalyzed only including participants in the Psychological condition agreeing in the inability item that the protagonist was literally unable to pick up Brown (*n* = 13). Of these participants, 92% selected the “Obligated but Unable” response, which exceeded chance rates, binomial test, *p* < .00001, test proportion = .25.

### Discussion

The overwhelming majority of participants judged that Walter is obligated to pick up Brown despite the physical or psychological inability to do so. Participants were much more likely to answer that suffering from a car accident made one “literally unable” to fulfill an obligation than suffering from clinical depression did. Participants were also significantly more likely to blame an agent for failing to fulfill an obligation when the reason for failure was described as psychological rather than physiological in nature. These asymmetries between physical and psychological conditions may be a manifestation of mental health stigma reflected by common misconceptions of mental illness (see General Discussion). For present purposes, however, we note that those participants answering “yes” to the inability item in the Psychological condition also overwhelmingly judged that Walter is obligated to pick up Brown. Experiment 1 serves as a powerful counterexample to OIC. But one objection is that the results are due to the specific wording of the vignettes or OIC test question. Given this, another experiment was conducted to replicate and expand the findings of Experiment 1. To do this, a completely different cover story was tested and probed for attributions of moral requirements using different moral terminology.

## Experiment 2

Experiment 2 was conducted to replicate and expand the results of Experiment 1 using a different cover story and different terminology to probe for the presence of moral requirements, including “duties” and “oughts”.

### Method

#### Participants

One hundred and sixty participants were tested (aged 18–72 years, mean age = 30.76 years; 93% reporting English as a native language; 62 female).

#### Materials and procedure

Participants were randomly assigned to one of four conditions in a 2 (Inability Type: Physical/Psychological) x 2 (Phrasing: Duty/Ought) experimental design. The stories all feature a playground safety worker named Michael, who sees some broken glass in an area where kids sometimes play barefoot [58]. In each story, Michael lacks either the physical or psychological ability to pick up the glass:

Michael is a playground safety worker. He sees some broken glass in an area where kids sometimes play barefoot. But he is stricken by a sudden [paralysis in his legs/panic attack]. As a result, Michael is not [physically/psychologically] able to pick up the glass.

After reading one of these stories, participants were given an OIC test question similar to the one used in Experiment 1 asking them select whether Michael is obligated and able to pick up the glass. However the four response options differed in the way that the requirement was phrased. Some participants was asked about what Michael “ought” to do (i.e. “Michael ought to pick up the glass”), while others were asked about what Michael has a “duty” to do (i.e. “Michael has a duty to pick up the glass”) despite having or lacking the physical or psychological ability to do so.

After answering this question, participants were taken to a new page and presented with the inability probe “Michael is literally unable to pick up the glass.” They were also again asked to rate their agreement with the statement “Michael deserves to be blamed for the fact that the glass was not picked up.” The scales and procedures of this experiment were identical to Experiment 1.

### Results

**Fig 1:**
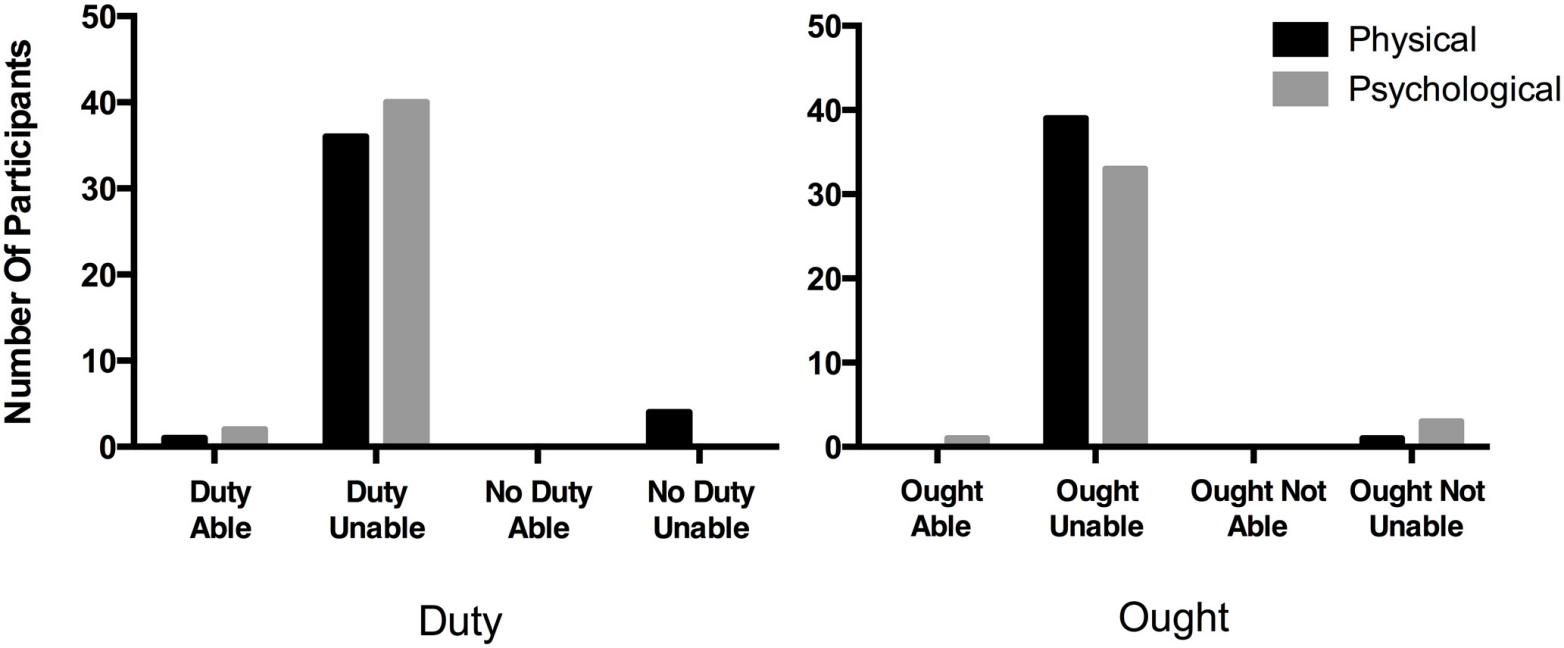
Attributions of Duty and Ought with Inability. The number of participants choosing each OIC test option for “duty” phrasing (left panel) and “ought” phrasing (right panel) across the Physical and Psychological conditions.

Multinomial logistic regression revealed that neither the Inability Type nor the Phrasing significantly affected answers in the OIC test question. In conditions adopting the “ought” phrasing, participants selected the “Ought but Unable” response for both Physical and Psychological conditions, 98%/89%, which each exceeded chance rates, binomial test, *p*s < .000001, test proportion = .25. In conditions adopting the “duty” phrasing, participants selected the “Duty but Unable” response for both Physical and Psychological conditions, 88%/95%, which each exceeded chance rates, binomial test, *p*s < .000001, test proportion = .25 (Fig 1).

Binary logistic regression analysis was conducted to predict response to the inability probe (“Michael is literally unable to pick up the glass”) using Inability Type and Phrasing as predictors. The model was statistically significant, X^2^ (2, *N* = 160) = 38.19, *p* < .000001, and explained between 21% and 34% of the variance in response to the inability probe. Responses to the inability probe were significantly predicted by Inability Type (Wald = 18.25, *p* < .0001), with 95/100% answering “yes” in the Physical Duty/Ought conditions, and 71/51% answering “yes” in the Psychological Duty/Ought conditions. By changing the type of inability from psychological to physical, the odds of judging the agent “literally unable” increased 25.29 times, 95% CI = 5.75 to 111.30. Phrasing was not a significant predictor.

A two-way analysis of variance yielded a large main effect of Inability Type on how much blame participants thought the protagonist deserves, *F*(1, 156) = 43.06, *p* < .000001, η_p_^2^ = .216. Blame scores were again significantly lower in Physical conditions, Duty (*M* = 2.32, *SD* = 1.64), Ought (*M* = 2.18, *SD* = 1.45) than in Psychological conditions, Duty (*M* = 4.10, *SD* = 1.90), Ought (*M* = 4.11, *SD* = 2.12). There were no other significant main or interaction effects.

To further evaluate OIC, we again reanalyzed responses to the OIC test question only including participants in Psychological Duty/Ought conditions answering “yes” to the inability item (*n* = 49). These participants selected the “Duty/Ought but Unable” responses, 100%/84%, which each exceeded chance rates, binomial test, *p*s < .000001, test proportion = .25.

### Discussion

The results replicated each of the three effects found in Experiment 1. The overwhelming majority of participants judged that Michael has a “duty” and “ought” to pick up the glass despite the physical or psychological inability to do so. We also continued to find differences between perceptions of physical and psychological inabilities. Participants were much more likely to answer that suffering from paralysis made one “literally unable” to fulfill moral requirements and less blameworthy for failing to fulfill them than suffering from panic attacks did. Participants were also significantly more likely to blame an agent for failing to act because of psychological inability. Those participants answering “yes” to the inability item in Psychological conditions again overwhelmingly judged that Michael ought and has a duty to pick up the glass.

## Experiment 3

Experiments 1 and 2 demonstrate that people think obligations, duties, and oughts persist despite an inability to fulfill them. But one objection to these studies is that they are limited to stories framed in third-person points of view. Prior work has shown that framing a problem from a certain perspective or point of view can influence cognitive evaluations [59, 60] and that ascriptions of moral responsibility are also susceptible to this effect [61]. This suggests that different patterns of responses might emerge when people consider the scenario first-personally. The present experiment tests this possibility.

### Method

#### Participants

One hundred and sixty-four participants were tested (aged 18–69 years, mean age = 34.27 years; 93% reporting English as a native language; 70 female).

#### Materials and procedure

Participants were randomly assigned to one of four conditions in a 2 (Inability Type: Physical/Psychological) x 2 (Perspective: Actor/Observer) between-subjects design.

Participants in each condition read a single story based on the airport vignettes administered in Experiment 1. Participants in the Observer conditions saw Physical or Psychological cases from Experiment 1 verbatim. Participants in the Actor conditions saw Physical or Psychological cases adapted as follows:

You promised that you would pick up Brown from the airport. But on the day of Brown’s flight, you are [in a serious car accident/suffering from clinical depression]. As a result, you are not [physically/psychologically] able to pick up Brown at the airport.

After reading a story, participants in the Observer conditions were presented with the same questions as participants in Experiment 1, adapted where appropriate for the actor tense (i.e. “You are obligated to pick up Brown at the airport, but you are not physically/psychologically able to do so.”) Participants in these conditions were also presented with an adapted inability item, “You are literally unable to pick up Brown at the airport” and blame item, “You deserve to be blamed for the fact that Brown was not picked up” using the same scales and procedures as Experiment 1.

### Results

A preliminary multinomial logistic regression revealed that response to the OIC test question was not significantly predicted by Inability Type, but it was predicted by Perspective. To explore this effect further, results were collapsed across the Inability Type condition and a chi-squared test was used to compare the rate at which participants selected the “Obligated but Unable” response. Participants in Actor conditions were more likely to select this response than participants in Observer conditions, 89/76%, X^2^ (1, *N* = 164) = 5.07, *p* < .05, Cramer’s V = .176.

**Fig 2:**
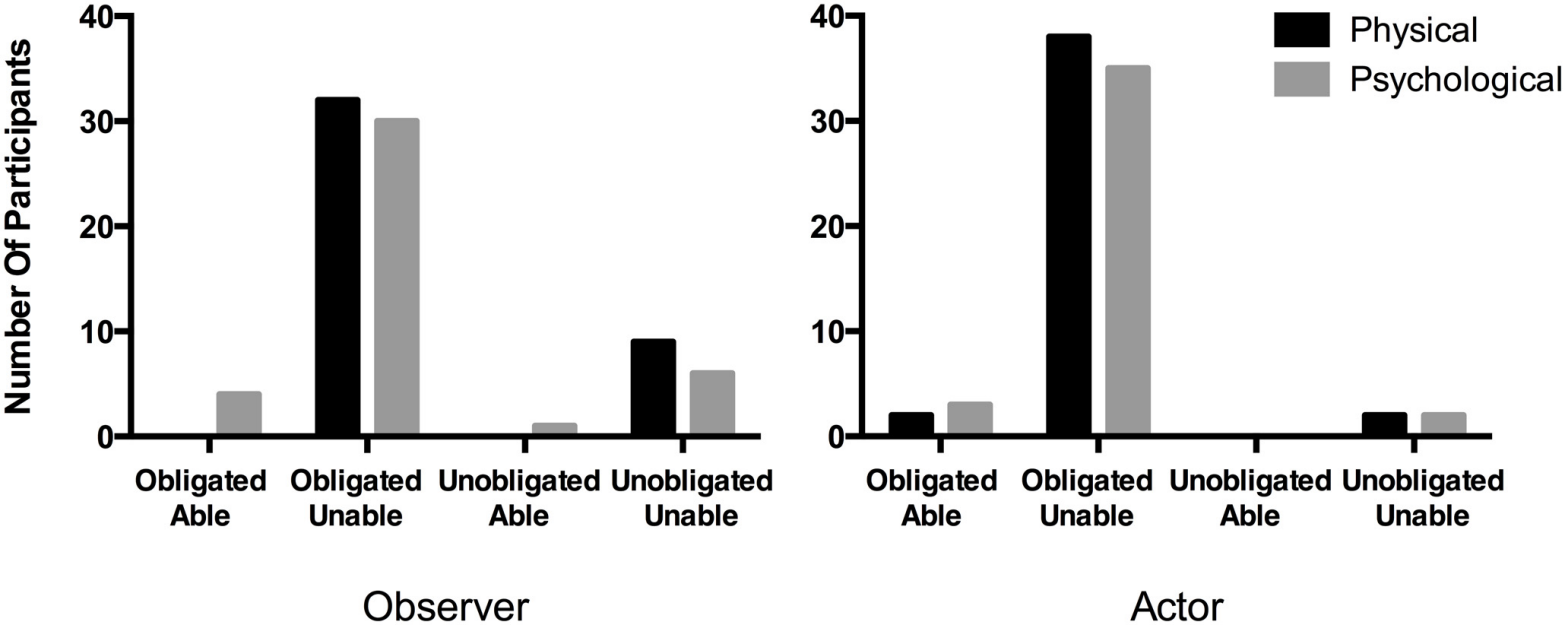
Attributions of Obligation with Inability to Observers and Actors. The number of participants choosing each OIC test option as best applying for Observer conditions (left panel) and Actor conditions (right panel) across Physical and Psychological conditions.

Despite this effect, participants selected the “Obligated but Unable” response to the OIC test question for all conditions of the experiment, Physical Observer, 78%; Physical Actor, 91%; Psychological Observer, 73%; Psychological Actor, 88%, which each exceeded chance rates, binomial test, *p*s < .000001, test proportion = .25 (Fig 2).

Binary logistic regression analysis was conducted to predict response to the inability probe (“Walter is/You are literally unable to pick up Brown at the airport”) using Inability Type and Perspective as predictors. The model was statistically significant, X^2^ (2, *N* = 164) = 68.91, *p* < .000001, and explained between 34% and 50% of the variance in response to the inability probe. Responses to the inability probe were significantly predicted by Inability Type (Wald = 19.51, *p* < .0001), with 100/98% answering “yes” in the Physical Observer/Actor conditions, and 51/43% answering “yes” in the Psychological Observer/Actor conditions. By changing the type of inability from psychological to physical, the odds of judging the agent “literally unable” increased 95.52 times, 95% CI = 12.63 to 722.44. Perspective was not a significant predictor.

A two-way analysis of variance yielded a large main effect for Inability Type on how much blame participants thought the protagonist deserves, *F*(1, 160) = 65.92, *p* < .000001, η_p_^2^ = .292. Blame scores were again significantly lower in Physical conditions, Observer (*M* = 1.59, *SD* = 1.02), Actor (*M* = 2.43, *SD* = 1.56), than in Psychological conditions, Observer (*M* = 3.78, *SD* = 1.78), Actor (*M* = 4.23, *SD* = 1.80). A two-way analysis of variance also yielded a small significant main effect for Perspective on blame scores, *F*(1, 160) = 6.86, *p* = .01, η_p_^2^ = .041. People were more likely to blame themselves for failing to fulfill obligations than they were to blame others. There were no interaction effects.

To further evaluate OIC, responses to the OIC test question were again reanalyzed only including participants in Psychological conditions answering “yes” to the inability item (*n* = 38). These participants selected the “Obligated but Unable” response in both Observer and Actor conditions, 62%/77%, which each exceeded chance rates, binomial test, *p*s < .001, test proportion = .25.

### Discussion

People were again likely to judge that obligations persist despite inability when evaluated from the third-person and first-person perspectives. Participants were also more likely to blame themselves than they were to blame others.

## Experiment 4

Prior experiments demonstrate that participants attribute a series of moral requirements to agents after they are rendered unable to act. However an important limitation of these studies is that in each case, the agent had an ability to act that was then later removed in the story due to a physical or psychological ailment. This increases the complexity of the judgments required and could potentially contribute to confusion in responses. Subsequent experiments address this concern by featuring cases that do not introduce inability in this way. Another limitation is that the experiments thus far have involved breaking a promise or preventing non-lethal physical harm. Will the same patterns of responses persist in cases with more serious moral requirements? This experiment was conducted to test whether moral requirements persist in the absence of ability in cases involving life-or-death circumstances. It also features a case where the agent faces a specific situation in which they are unable to act due to uncontrollable features of that situation, rather than through the introduction of a personal ailment rendering them able or unable to act at different times in the story.

### Method

#### Participants

Forty-one participants were tested (aged 18–70 years, mean age = 34.76 years; 93% reporting English as a native language; 26 female).

#### Materials and procedure

Participants were assigned to one condition:

Jessica is a lifeguard at a remote ocean beach. Two struggling swimmers are about to drown. Jessica rushes in to save them. But because of the very far distance between the swimmers, it is physically impossible for her to rescue both swimmers. Jessica rescues the one swimmer but not the other.

Participants were presented with a similar OIC test question used in prior experiments. This time participants were asked whether Jessica had or did not have a duty to rescue both swimmers, on the one hand, along with whether she was physically or not physically able to do so, on the other. Participants were also presented with an adapted inability item, “Jessica is literally unable to rescue both swimmers” and blame item, “Jessica deserves to be blamed for the fact that a swimmer drowns” using the same scales and procedures as Experiments 1–3.

### Results

Ninety-three percent of participants gave the “Duty but Unable” answer, which exceeded chance rates binomial test, *p* < .000001, test proportion = .25. Ninety-five percent of participants answered that Jessica was literally unable to rescue both swimmers, which exceeded chance rates, binomial test, *p* < .000001, test proportion = .5. Participants also strongly disagreed that Jessica deserved to be blamed for the fact that a swimmer drowns (*M* = 1.83, *SD* = 1.14). Blame scores were significantly below the neutral midpoint of 4, *t*(40) = -12.21, *p* < .000001.

### Discussion

The overwhelming majority of participants attribute moral requirements despite physical inability when the consequences of failure were severe. These results also show that this pattern persists for very different sorts of physical inability. The vignettes in Experiments 1–3 featured physical inabilities involving factors “inside” the agent, due to disablement of an agent’s personal force, such as paralysis or the use of one’s legs. In the present experiment similar results were observed when an agent’s physical inability results from situational factors “outside” the agent, such as opportunity and physical location. Participants ascribe duties to the agent even though the reason why Jessica is unable to save both swimmers is because of uncontrollable features of the situation, rather than through the introduction of an ailment rendering her able to save both drowning swimmers at one time, and unable to do so at another time.

Though participants strongly attribute duties, it might be objected that they only do so in this and prior experiments because the protagonists make a prior commitment or occupy a prior role before an inability is introduced in the story. It also might still be objected that participants in prior studies conceive of oughts as applying to agents only before the introduction of an inability. The following experiment tests these possibilities.

## Experiment 5

This experiment tests whether ought attributions persist despite inability in the absence of a prior commitment or role, such as having made a promise or being a lifeguard. It also tests whether these attributions continue when the duration of the inability is both recently introduced or is the result of a lifelong inability.

### Method

#### Participants

Eighty-one participants were tested (aged 19–59 years, mean age = 30.74 years; 96% reporting English as a native language; 27 female).

#### Materials and procedure

Participants were randomly assigned to one of two conditions featuring an agent with an inability to act and who also occupies no explicit prior commitment or moral role. The conditions differed in whether that inability was recent or lifelong:

Michael is relaxing in the park when he sees a small girl fall into a nearby pond. She is drowning and definitely will die unless someone quickly pulls her out. This part of the park is secluded and Michael is the only person around. But [Michael is stricken by a sudden paralysis in his legs/Michael's legs have been paralyzed since birth]. As a result, Michael is not physically able to save the girl.

Following past experiments, participants were then asked to “choose the option that best applies” from the list below:

1. Michael ought to save the girl, and Michael is physically able to do so.
2. Michael ought to save the girl, but Michael is not physically able to do so.
3. Michael ought not save the girl, but Michael is physically able to do so.
4. Michael ought not save the girl, and Michael is not physically able to do so.

Participants were also presented with an adapted inability item, “Michael is literally unable to save the girl” and blame item, “Michael deserves to be blamed for the fact that the girl drowns” using the same scales and procedures as prior experiments.

### Results

Assignment to condition did not affect responses to the OIC test question, X^2^ (3, *N* = 81) = 4.38, *p* = .224. Participants selected the “Ought but Unable” response for both Recent and (88%) Lifelong (95%) conditions at rates exceeding chance, binomial test, *p*s < .000001, test proportion = .25. Assignment to condition did not affect whether participants thought Michael was “literally unable” to save the girl, 95/95%, X^2^ (1, *N* = 81) = 0.001, *p* = .98. Participants in both conditions strongly disagreed that Michael deserved to be blamed for the fact that the girl drowns (*M*s = 2.05/1.38, *SD*s = 1.36/0.63), though they were more willing to blame the agent for failure when the inability was recent than when it was lifelong, *t*(79) = 2.85, *p* < .01, η_p_^2^ = .093.

### Follow-up

It might be objected that the results from Experiment 5 were partly due to the oddness of having options that say Michael “ought not” to save the girl. We believe that this issue is satisfactorily addressed by the results of Experiment 2, where we observed no difference between probes using “ought not” and “does not have a duty.” That is, in light of the earlier findings, it is unlikely that the results in Experiment 5 were driven by any oddness associated with “ought not” wording. However, out of an abundance of caution, we ran a follow-up that used the exact same design and cover story as Experiment 5, but which used different response options for the OIC test question:

1. Michael has a duty to save the girl, and he is physically able to do so.
2. Michael has a duty to save the girl, but he is not physically able to do so.
3. Michael does not have a duty to save the girl, but he is physically able to do so.
4. Michael does not have a duty to save the girl, and he is not physically able to do so.

Eighty new participants (aged 18–63, mean age = 31.55; 95% reporting English as a native language; 31 female) were tested. Assignment to condition did not affect response to the OIC test question, X^2^ (2, *N* = 80) = 0.13, *p* = .939. Participants selected the “Has a Duty but Is Unable” response for both Recent and (88%) Lifelong (85%) conditions at rates exceeding chance, binomial test, *p*s < .000001, test proportion = .25. These results conclusively rule out the objection that the findings in Experiment 5 are due to oddness associated with “ought not” wording.

### Discussion

Participants again ascribed oughts in tandem with inability in conditions where no prior moral commitments or roles are stated. Nearly all participants judged that agents in these conditions ought to act despite the onset of recent or lifelong inability to do so. Moreover, it might have been suggested that participants ascribe oughts in prior experiments only because they focused on a time before an inability was introduced in the story. This experiment undermines that interpretation because oughts were attributed to agents with lifelong inabilities who were at no time able to act.

## Experiment 6

Experiment 5 shows that participants are insensitive to the duration of an inability when they make judgments about what an agent ought to do. Nonetheless, these cases still feature inabilities that differentially affect the performance of a particular agent over others. Perhaps we would observe a different pattern of results if the scope of the inability was not limited to one particular agent, but rather, more generally prevented all agents from acting. This experiment tests whether the scope of the inability affects ought ascriptions.

### Method

#### Participants, materials and procedure

Eighty-one participants were tested (aged 18–66 years, mean age = 29.50 years; 93% reporting English as a native language; 31 female). Participants were randomly assigned to one of the two conditions presented in Experiment 5. The only difference in materials was that the cases were adapted such that the inability to act was either specific to a particular agent or general to all humans:

Michael is relaxing in the park when he sees a small girl fall into a nearby pond. She is drowning and definitely will die unless someone quickly pulls her out. This part of the park is secluded and Michael is the only person around. But [Michael cannot/no human could] swim fast enough to save the girl. As a result, Michael is not physically able to save the girl.

Participants answered the same questions using the same procedure as Experiment 5.

### Results

Assignment to condition did not affect responses to the OIC test question, X^2^ (3, *N* = 81) = 3.44, *p* = .328. Participants selected the “Ought but Unable” response for both Particular (90%) and General (95%) conditions at rates exceeding chance, binomial test, *p*s < .000001, test proportion = .25. Assignment to condition did not affect whether participants thought Michael was “literally unable” to save the girl, 80/90%, X^2^ (1, *N* = 81) = 1.68, *p* = .194, or how much blame Michael deserves for the fact that the girl drowns (*M*s = 2.20/1.78, *SD*s = 1.62/0.99), *t*(79) = 1.41, *p* = .162.

### Discussion

Participants were insensitive to ability when they made judgments about what an agent ought to do, even in cases where no human beings ever have the ability to perform the relevant action.

## Experiment 7

Participants attribute requirements in tandem with physical inability in a wide variety of circumstances. But perhaps participants are ascribing obligations, duties and “oughts” in these situations indiscriminately, in ways that do not truly reflect their moral concepts. To test this possibility, we collected obligation attributions in cases where ability is present or absent while also distinguishing *moral* obligation from another familiar form of obligation: *legal* obligation.

### Method

#### Participants

One hundred and sixty participants were tested (aged 18–73 years, mean age = 33.20 years; 94% reporting English as a native language; 62 female).

#### Materials and procedure

Participants were randomly assigned to one of four conditions in a 2 (Ability: Unable/Able) x 2 (Obligation Type: Legal/Moral) experimental design. The stories all featured the same basic text used in Experiment 6. The stories were adapted such that Michael either has or lacks the ability to act as follows:

Michael is relaxing in the park when he sees a small girl fall into a nearby pond. She is drowning and definitely will die unless someone quickly pulls her out. This part of the park is secluded and Michael is the only person around. [But Michael is stricken with a sudden paralysis in his legs and cannot swim to save the girl. As a result, Michael is not physically able to save the girl./And Michael is a normal adult male and can swim fast enough to save the girl. As a result, Michael is physically able to save the girl.]

After reading one of these stories, participants were given an OIC test question similar to the one used in prior experiments. However the response options featured different types of obligation. One group of participants was asked about “legal” obligation (i.e. “Michael is legally obligated…”), while another group were asked about what Michael has a “moral” obligation to do. Participants answered the same subsequent questions using the same procedure as Experiment 6.

### Results

Preliminary multinomial logistic regression analysis revealed that responses to the OIC test question were significantly predicted by Ability and Obligation Type. However, some response options went unselected in some conditions, and logistic models are not recommended when this happens [62]. Hence, to explore this effect, we conducted pairwise comparisons using chi-squared tests.

**Fig 3:**
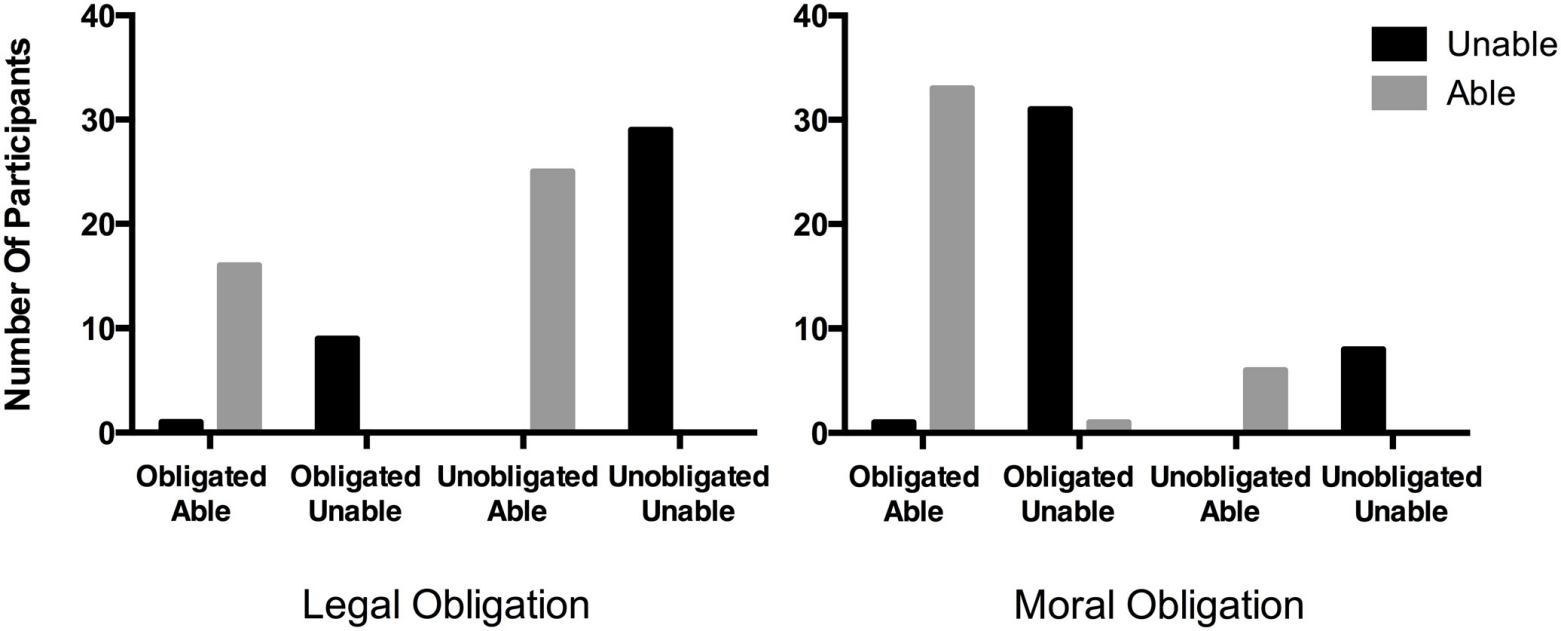
The Role of Ability in Moral and Legal Obligations. The number of participants choosing each OIC test option as best applying for Legal conditions (left panel) and Moral conditions (right panel) across Unable and Able conditions.

Pairwise comparisons reveal that Ability affected responses to the OIC test question for both Legal, X^2^ (3, *N* = 80) = 76.23, *p* < .000001, Cramer’s V = .98, and Moral conditions, X^2^ (3, *N* = 80) = 72.24, *p* < .000001, Cramer’s V = .95. Pairwise comparisons also reveal that Obligation Type affected responses to the OIC test question in both Unable conditions, X^2^ (2, *N* = 79) = 24.01, *p* < .00001, Cramer’s V = .55, and Able conditions, X^2^ (2, *N* = 81) = 18.53, *p* < .0001, Cramer’s V = .48 (Fig 3).

In the Legal Able condition participants selected the options “obligated and able” (39%) and “unobligated but able” (61%) at rates exceeding chance, binomial test, *p*s < .05, test proportion = .25. In the Legal Unable condition 74% of participants selected the “unobligated and unable” response, which exceeded chance rates, binomial test, *p* < .000001, test proportion = .25. In the Moral Able condition 83% of participants selected the “obligated and able” response, which exceeded chance rates, binomial test, *p* < .000001, test proportion = .25. In the Moral Unable condition 78% of participants selected the “obligated but unable” which exceeded chance rates, binomial test, *p* < .000001, test proportion = .25.

Binary logistic regression analysis was conducted to predict response to the inability probe (“Michael is literally unable to save the girl”) using Ability and Obligation Type as predictors. The model was statistically significant, X^2^ (2, *N* = 160) = 171.37, *p* < .000001, and explained between 66% and 88% of the variance in response to the inability probe. Responses to the inability probe were significantly predicted by Ability (Wald = 56.26, *p* < .000001), with 0% and 8% answering “yes” in the Legal and Moral Unable conditions, compared to 97% in both the Legal and Moral Able conditions, odds ratio 740.5, 95% CI = 131.73 to 4162.50. Obligation type was not a significant predictor.

A two-way analysis of variance yielded a large main effect of Ability on how much blame participants thought the protagonist deserves, *F*(1,156) = 80.47, *p* < .000001, η_p_^2^ = .34. Blame scores were again significantly lower in Unable conditions, Legal (*M* = 1.85, *SD* = 1.14), Moral (*M* = 2.03, *SD* = 1.39), than in Able conditions, Legal (*M* = 4.59, *SD* = 1.91), Moral (*M* = 4.05, *SD* = 2.09). There were no other significant main or interaction effects.

### Discussion

Moral obligations are continually ascribed in tandem with inability, even though another familiar form of obligation does not exist in the same exact scenario. This suggests that participants are applying their concepts of moral and legal obligation in these scenarios discriminately, even though they may have different ideas about the presence or absence of obligations in these two domains.

## Experiment 8

Results from the earlier experiments demonstrated that commonsense morality does not endorse OIC. But the results also suggested a close connection between ability and blame judgments. Thus, one might be led to wonder, even though ought does not imply can, perhaps blame does. This experiment was designed as an initial test of this possibility. The research question is whether ascriptions of ability and blame are consistent with a “blame implies can” principle, or if commonsense morality rejects this as readily as an “ought implies can” principle.

### Method

#### Participants

Seventy-nine participants were tested (aged 18–56 years, mean age = 31.30 years; 98% reporting English as a native language; 31 females).

#### Materials and procedure

Participants were randomly assigned to one of two conditions (Obligation and Blame) in a between-subjects design. Participants in both conditions read the same story that was administered in the physical condition of Experiment 1, in which Walter is physically unable to pick up Brown. After reading this story, one group of participants was given the OIC test question from Experiment 1, while the other group was given a “blame implies can” (hereafter BIC) test question. The BIC test question asked participants to select the best option from the responses below:

1. Walter is blameworthy for not picking up Brown at the airport, and Walter is physically able to pick him up.
2. Walter is blameworthy for not picking up Brown at the airport, but Walter is not physically able to pick him up.
3. Walter is not blameworthy for not picking up Brown at the airport, but Walter is physically able to pick him up.
4. Walter is not blameworthy for not picking up Brown at the airport, and Walter is not physically able to pick him up.

Participants in both conditions answered the same two subsequent questions using the same procedure as Experiment 1.

### Results

**Fig 4:**
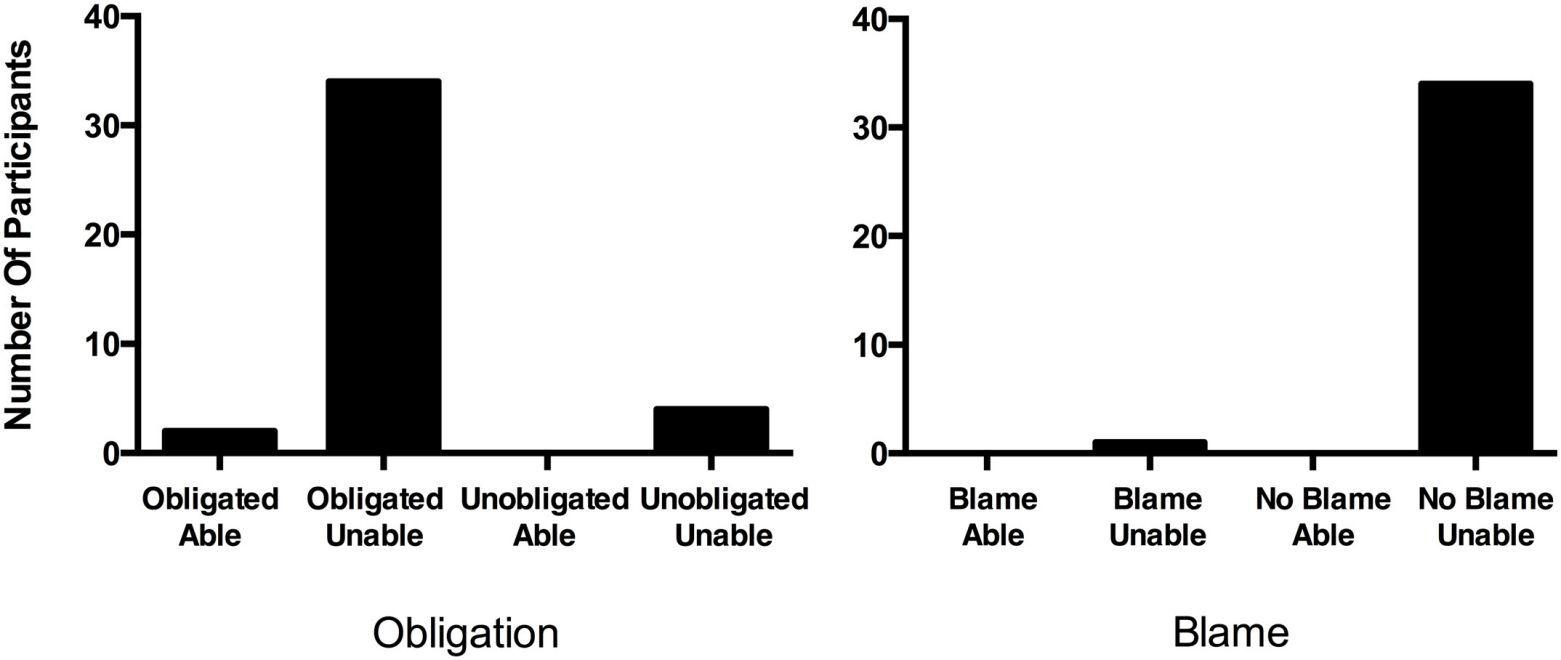
The Role of Ability in Attributions of Obligation and Blame. The number of participants choosing each OIC test option (left panel) and each BIC test option (right panel) in Obligation and Blame conditions.

Responses were significantly different between OIC and BIC test questions, X^2^ (2, N = 79) = 60.64, *p* < .000001, Cramer’s V = .876. Eighty-five percent of participants selected the “Ought but Unable” response to the OIC test question, which exceeds chance rates, binomial test, *p* < .000001, test proportion = .25. By contrast, 97% percent of participants selected the “Not blameworthy and Unable” response to the BIC test question, which exceeds chance rates, binomial test, *p* < .000001, test proportion = .25 (Fig 4). Assignment to OIC or BIC condition did not affect whether participants thought Walter was “literally unable” to pick up Brown (both 100%), or how much blame Walter deserves for not picking up Brown (*M*s = 1.75/1.69, *SD*s = 0.90/1.30), *t*(77) = 0.23, *p* = .819.

### Discussion

The results provide initial support for the hypothesis that unlike obligations, blameworthiness is not ascribed independently of perceptions of ability. This suggests that although ordinary moral cognition does not endorse an “ought implies can” principle, it may support a closely related principle, namely, “blame implies can.” In fact, we found that attributions of ability and blame fit the exact pattern that “ought implies can” predicts regarding ability and obligation. We emphasize that this experiment was intended only as an initial test. We do not firmly conclude that commonsense firmly endorses a “blame implies can” principle. Future research is needed to evaluate the extent to which ability and blame are related in the context of moral judgment beyond the patterns suggested here.

## Conclusions

Commonsense moral cognition rejects the principle that ought implies can. Experiment 1 showed that obligations persist despite the inability to fulfill them. Experiment 2 generalized this finding to other narrative contexts and using different vocabulary to probe for obligation attributions. Experiment 3 generalized the finding to third-person and first-person evaluations. Experiment 4 generalized the finding to cases involving serious moral obligations in life-or-death circumstances. Experiments 5–6 demonstrated that this pattern of judgments is not diminished when alternating the duration or scope of inability. Experiment 7 shows that obligations are not ascribed indiscriminately. Moral obligations persist with or without ability even though other obligations, such as legal obligations are not attributed in the same circumstances. Lastly, Experiment 8 suggests that, unlike attributions of obligation, attributions of blame are informed by considerations of ability, which lends support to the related principle that blame implies can.

We used a variety of different moral terminology to evaluate whether commonsense morality is inclined to accept an “ought implies can” principle, or something thereabout, including “ought”, “obligation”, and “duty”. One could potentially draw many subtle philosophical distinctions among these concepts, and in principle they each might have related to ability differently in ordinary moral judgment. We found that each was attributed independently of ability.

In Experiments 1–3 we also saw important differences between the way physical and psychological inabilities were perceived. Such differences between perceptions of psychological and physical inability reveal a disquieting picture of the ordinary perception of psychological disorders. These results cohere with prior research on mental health stigma, whereby mental illness is viewed as the result of “personal weakness” or lack of “self-control” [63–65] and as the result of “dualistic biases” among laypeople and mental health professionals that contribute to a growing crisis in modern psychiatric treatment [66]. Our results indicate that although obligations persist equally in both cases, psychologically incapable agents are more likely to be perceived as able to fulfill their obligations, and more likely to be blamed for failing to fulfill those obligations, than their counterparts with analogous physical inabilities.

These results highlight an important conceptual distinction between moral obligation and blame. It has long been thought that to be morally obligated to do something is to be blameworthy or properly criticizable for failing to do it [67]. Our results strongly rule out this possibility. Attributions of moral obligation were insensitive to whether the agent was considered blameworthy for failing to achieve it. In Experiment 7, for instance, the overwhelming majority of participants agreed that a bystander has a moral obligation to rescue a drowning child, but they strongly disagreed that the bystander is blameworthy for the fact that the child drowns when they are unable to prevent it. These results suggest that in ordinary moral cognition there is an important difference between recognizing and attributing obligations, on the one hand, and evaluating unfulfilled obligations, on the other.

The findings may also fundamentally inform responses to moral dilemmas. It is a typical feature of many classic moral dilemmas—such as trolley problems [35, 68] or lifeguard cases (Experiment 4)—that agents must choose to fulfill one moral obligation at the expense of another. Our findings suggest that moral obligations persist despite the inability to fulfill them, in precisely those life-or-death circumstances characteristic of moral dilemmas. In other words, the rejection of “ought implies can” seems to speak to the very heart of why these situations strike us as so agonizing in the first place [32].

These findings also have broader implications for a series of questions at the intersection of criminal justice and jurisprudence. The law regularly makes allowances for agents who lack certain abilities, for instance the ability to control their actions or discern right from wrong. In some of these cases agents may be declared “not guilty by reason of insanity.” Insanity defenses are not as common as one might think, and successful insanity defenses are often controversial [69, 70]. The judgment that moral obligations persist even when legal obligations do not could explain mixed reactions to insanity verdicts. In other words, nullifying legal obligations on the basis of inability might seem counterintuitive because people recognize that inability does not nullify corresponding moral obligations in the same circumstances.

Lastly, these results may also be relevant to the philosophical literature on the “ought implies can” principle. Though commonsense morality rejects “ought implies can,” many philosophers have defended it on the grounds that it is correct [8, 9, 12, 17]. Thus it would be satisfying to understand why the link between ability and obligation might have appeared intuitive to some. A large body of prior research has shown that the motivation to blame someone often leads people to distort causal facts and interpret them in a way that justifies their negative reactions, a phenomenon known as *blame validation* [71, 72]. And new research has shown a further dimension to this phenomenon, known as *excuse validation* [57, 73]. The motivation to exculpate often leads people to distort causal facts and interpret them in a way that justifies their emotional reaction. For example, when people recognize that someone has blamelessly broken a rule, this can lead them to claim, paradoxically, that no rule was broken at all. One hypothesis, then, is that the reported intuition that “ought implies can” is an instance of excuse validation: the desire to excuse agents for blamelessly failing to fulfill obligations has led some to deny that the obligations exist in the first place. Alternatively, but relatedly, perhaps some have sensed that “blame implies can” is an intuitively powerful principle, but mistook this intuition as evidence that “ought implies can.”

## References

[1] Mikhail J. Universal moral grammar: Theory, evidence, and the future. Trends in Cognitive Sciences. 2007;11(4):143–52. 17329147 10.1016/j.tics.2006.12.007

[2] Tomasello M, Vaish A. Origins of Human Cooperation and Morality. Annual Review of Psychology. 2013;64(1):231–55. 10.1146/annurev-psych-113011-143812 22804772

[3] Fehr E, Fischbacher U. Social norms and human cooperation. Trends in Cognitive Sciences. 2004;8(4):185–90. 10.1016/j.tics.2004.02.007 15050515

[4] Singer P. Famine, affluence, and morality. Philosophy and Public Affairs. 1972;1(3):229–43.

[5] Scheffler S. Morality's demands and their limits. Journal of Philosophy. 1986;83(10):531–7.

[6] Kagan S. Does consequentialism demand too much? Recent work on the limits of obligation. Philosophy and Public Affairs. 1984;13(3):239–54.

[7] Mulgan T. The Demands of Consequentialism: Oxford University Press; 2001 289–96 p.

[8] Cicero MT. Cicero's Three Books Of Offices, Or Moral Duties: Also His Cato Major, an Essay on Old Age; Laelius, an Essay on Friendship; Paradoxes; Scipio's Dream; and Letter to Quintus on the Duties of a Magistrate: H. G. Bohn; 1856.

[9] Kant I. Religion Within the Boundaries of Mere Reason and Other Writings: Cambridge University Press; 1998.

[10] Copp D. 'Ought' implies 'can' and the derivation of the principle of alternate possibilities. Analysis. 2008;68(297):67–75.

[11] Dahl NO. Ought implies can and deontic logic. Philosophia. 1974;4(4):485–511.

[12] Feldman F. Doing the Best We Can: An Essay in Informal Deontic Logic: D. Reidel Publishing Company; 1986 264–7 p.

[13] Flanagan OJ. Varieties of Moral Personality: Ethics and Psychological Realism: Harvard University Press; 1991 104 p.

[14] Hare RM. Freedom and Reason: Oxford, Clarendon Press; 1963 358 p.

[15] Howard-Snyder F. “Cannot” implies “not ought”. Philosophical Studies. 2006;130(2):233–46.

[16] Littlejohn C. Does 'Ought' Still Imply 'Can'? Philosophia. 2012;40(4):821–8.

[17] Moore GE. The nature of moral philosophy Philosophical papers: Routledge and Kegan Paul; 1922.

[18] Stern R. Does ‘ought’ imply ‘can’? And did Kant think it does? Utilitas. 2004;16(1):42–61.

[19] Streumer B. Does 'ought' conversationally implicate 'can'? European Journal of Philosophy. 2003;11(2):219–28.

[20] Van Fraassen BC. Values and the heart's command. Journal of Philosophy. 1973;70(1):5–19.

[21] Vranas PBM. I Ought, Therefore I Can. Philosophical Studies. 2007;136(2):167–216.

[22] Blum A. The Kantian versus Frankfurt. Analysis. 2000;60(3):287–8.

[23] Frankfurt HG. Alternate possibilities and moral responsibility. Journal of Philosophy. 1969;66(3):829–39.

[24] Frankfurt HG. What are we morally responsible for The Importance of What We Care About: Cambridge University Press; 1988 p. 95–113.

[25] Spencer J. What time travelers cannot not do (but are responsible for anyway). Philosophical Studies. 2013;166(1):149–62.

[26] Fischer JM. ‘Ought-implies-can’, causal determinism and moral responsibility. Analysis. 2003;63(279):244–50.

[27] van Inwagen P. An Essay on Free Will: Oxford University Press; 1983 401–8 p.

[28] Woolfolk RL, Doris JM, Darley JM. Identification, situational constraint, and social cognition: studies in the attribution of moral responsibility. Cognition. 2006;100(2):283–301. 10.1016/j.cognition.2005.05.002 . 16087171

[29] Conee E. Against moral dilemmas. Philosophical Review. 1982;91(1):87–97.

[30] Jacquette D. Moral dilemmas, disjunctive obligations, and Kant's principle that 'ought' implies 'can'. Synthese. 1991;88(1):43–55.

[31] Marcus RB. Moral dilemmas and consistency. Journal of Philosophy. 1980;77(3):121–36.

[32] Sinnott-Armstrong W. Moral Dilemmas: B. Blackwell; 1988 240 p.

[33] Ryan S. Doxastic compatibilism and the ethics of belief. Philosophical Studies. 2003;114(1–2):47–79.

[34] Greene JD. The Secret Joke of Kant's Soul In: Sinnott-Armstrong W, editor. Moral Psychology, Vol 3: The Neuroscience of Morality: Emotion, Disease, and Development. Cambridge, MA: MIT Press; 2007.

[35] Greene JD, Cushman FA, Stewart LE, Lowenberg K. Pushing moral buttons: The interaction between personal force and intention in moral judgment. Cognition. 2009;11(364–371). 10.1016/j.cognition.2009.02.001 19375075

[36] Thomson JJ. Rights, Restitution, and Risk: Essays, in Moral Theory: Harvard University Press; 1986 414–8 p.

[37] Cushman F. Crime and punishment: Distinguishing the roles of causal and intentional analyses in moral judgment. Cognition. 2008;108(2):353–80. 10.1016/j.cognition.2008.03.006 18439575

[38] Williams BAO. Moral Luck. Cambridge: Cambridge University Press; 1981.

[39] Guglielmo S, Malle BF. Can Unintended Side Effects Be Intentional? Resolving a Controversy Over Intentionality and Morality. Personality and Social Psychology Bulletin. 2010;36(12):1635–47. 10.1177/0146167210386733 21051767

[40] Knobe J. Person as scientist, person as moralist. Behavioral and Brain Sciences. 2010;33(04):315–29. 20964912 10.1017/S0140525X10000907

[41] Goodwin GP, Darley JM. The psychology of meta-ethics: exploring objectivism. Cognition. 2008;106(3):1339–66. Epub 2007/08/19. 10.1016/j.cognition.2007.06.007 . 17692306

[42] Bloom P. Descartes' baby: how the science of child development explains what makes us human. New York: Basic Books; 2004.

[43] Van de Vondervoort JW, Friedman O. Parallels in Preschoolers' and Adults' Judgments About Ownership Rights and Bodily Rights. Cognitive Science. 2015;39(1):184–98. 10.1111/cogs.12154 25066448

[44] Ajzen I. The theory of planned behavior. Organizational Behavior and Human Decision Processes. 1991;50(2):179–211. 10.1016/0749-5978(91)90020-T.

[45] Ajzen I. Perceived Behavioral Control, Self-Efficacy, Locus of Control, and the Theory of Planned Behavior1. Journal of Applied Social Psychology. 2002;32(4):665–83. 10.1111/j.1559-1816.2002.tb00236.x

[46] Bandura A, Adams N, Hardy A, Howells G. Tests of the generality of self-efficacy theory. Cogn Ther Res. 1980;4(1):39–66. 10.1007/BF01173354

[47] Bandura A. Self-efficacy mechanism in human agency. American Psychologist. 1982;37(2):122–47. 10.1037/0003-066X.37.2.122

[48] Brandts J, Solà C. Reference Points and Negative Reciprocity in Simple Sequential Games. Games and Economic Behavior. 2001;36(2):138–57. 10.1006/game.2000.0818.

[49] Nelson WR Jr. Equity or intention: it is the thought that counts. Journal of Economic Behavior & Organization. 2002;48(4):423–30. 10.1016/S0167-2681(01)00245-1.

[50] Falk A, Fehr E, Fischbacher U. On the Nature of Fair Behavior. Economic Inquiry. 2003;41(1):20–6. 10.1093/ei/41.1.20

[51] Sutter M. Outcomes versus intentions: On the nature of fair behavior and its development with age. Journal of Economic Psychology. 2007;28(1):69–78. 10.1016/j.joep.2006.09.001.

[52] Cushman F, Dreber A, Wang Y, Costa J. Accidental Outcomes Guide Punishment in a “Trembling Hand” Game. PLoS ONE. 2009;4(8):e6699 10.1371/journal.pone.0006699 19707578 PMC2726629

[53] Darley JM, Carlsmith KM, Robinson PH. Incapacitation and just deserts as motives for punishment. Law Hum Behav. 2000;24(6):659–83. Epub 2000/12/06. . 11105478 10.1023/a:1005552203727

[54] Robinson P, Darley JM. Justice, Liability And Blame: Community Views And The Criminal Law. Westview Press; 1996.

[55] Driver J. Promises, obligations, and abilities. Philosophical Studies. 1983;44(2):221–3.

[56] Sinnott-Armstrong W. `Ought' conversationally implies `can'. Philosophical Review. 1984;93(2):249–61.

[57] Turri J, Blouw P. Excuse validation: a study in rule-breaking. Philosophical Studies. 2015;172(3):615–34.

[58] Stocker M. 'Ought' and 'can'. Australasian Journal of Philosophy. 1971;49(3):303–16.

[59] Jones EE, Nisbett RE. The actor and the observer: Divergent perceptions of the causes of behavior. Genearl Learning Press; 1971.

[60] Choi I, Nisbett RE. Situational Salience and Cultural Differences in the Correspondence Bias and Actor-Observer Bias. Personality and Social Psychology Bulletin. 1998;24(9):949–60. 10.1177/0146167298249003

[61] Tobia K, Buckwalter W, Stich S. Moral intuitions: Are philosophers experts? Philosophical Psychology. 2013;26(5):629–38.

[62] Garson G. Testing Statistical Assumptions. Statistical Associates Publishing 2012.

[63] Byrne P. Stigma of mental illness and ways of diminishing it. Advances in Psychiatric Treatment. 2000;6(1):65–72.

[64] Hinshaw SP, Stier A. Stigma as Related to Mental Disorders. Annual Review of Clinical Psychology. 2008;4(1):367–93. 10.1146/annurev.clinpsy.4.022007.141245 17716044

[65] Weiner B, Perry RP, Magnusson J. An attributional analysis of reactions to stigmas. J Pers Soc Psychol. 1988;55(5):738–48. Epub 1988/11/01. . 2974883 10.1037//0022-3514.55.5.738

[66] Miresco MJ, Kirmayer LJ. The persistence of mind-brain dualism in psychiatric reasoning about clinical scenarios. Am J Psychiatry. 2006;163(5):913–8. Epub 2006/05/02. . 16648335 10.1176/ajp.2006.163.5.913

[67] Mill JS. Utilitarianism. Sher G, editor. Indianapolis: Hackett; 1979.

[68] Millar JC, Turri J, Friedman O. For the greater goods? Ownership rights and utilitarian moral judgment. Cognition. 2014;133(1):79–84. 10.1016/j.cognition.2014.05.018. 10.1016/j.cognition.2014.05.018 24972369

[69] Hans VP. An Analysis of Public Attitudes Toward the Insanity Defense. Criminology. 1986;24(2):393–414. 10.1111/j.1745-9125.1986.tb01502.x

[70] Sinnott-Armstrong W, Levy K. Insanity defense In: Deigh J, Dolinko D, editors. The Oxford Handbook of the Philosophy of the Criminal Law: Oxford University Press; 2011 p. 299–334.

[71] Alicke MD. Culpable control and the psychology of blame. Psychological Bulletin. 2000;126(4):556–74. 10900996 10.1037/0033-2909.126.4.556

[72] Alicke MD, Buckingham J, Zell E, Davis T. Culpable Control and Counterfactual Reasoning in the Psychology of Blame. Personality and Social Psychology Bulletin. 2008;34(10):1371–81. 10.1177/0146167208321594 18644856

[73] Turri J. The test of truth: An experimental investigation of the norm of assertion. Cognition. 2013;129(2):279–91. 10.1016/j.cognition.2013.06.012 23954823

